# Beyond the Six-Week Rule: Contemporary Insights Into Pancreatic Pseudocyst

**DOI:** 10.7759/cureus.110750

**Published:** 2026-06-12

**Authors:** Nikunj Barvadiya, Divyeshkumar N Parmar, Minesh Sindhal, Divyakant Barot, Priyanka Aanandaka, Anjali M Aghera, Nidhi Gheewala, Nancy Solanki, Mohith VS, Anand Kumar

**Affiliations:** 1 General Surgery, All India Institute of Medical Sciences (AIIMS), Rajkot, Rajkot, IND; 2 Surgery, All India Institute of Medical Sciences (AIIMS), Rajkot, Rajkot, IND

**Keywords:** cystic pancreatic neoplasm, intraductal papillary mucinous neoplasm (ipmn), pancreatic pseudocyst, pancreatitis chronic, severe pancreatitis

## Abstract

Pancreatic pseudocysts are a frequent consequence of pancreatic injury and represent a heterogeneous group of fluid collections with variable clinical behavior. While some resolve spontaneously, others may lead to complications such as infection, hemorrhage, or obstruction, often after a prolonged and uncertain clinical course. Advances in cross-sectional imaging and endoscopic ultrasound have improved the ability to distinguish pseudocysts from other pancreatic fluid collections and cystic lesions, enabling more accurate diagnosis and safer intervention. At the same time, minimally invasive endoscopic and image-guided techniques have significantly reduced the need for surgical management in appropriately selected patients.

This review provides a practical clinical overview of pancreatic pseudocysts, focusing on pathophysiology, diagnostic challenges, and treatment strategies. Emphasis is placed on a stepwise, individualized approach in which conservative management, endoscopic drainage, percutaneous techniques, and surgery each have defined roles. Effective management depends on clinical presentation, complications, ductal anatomy, and the timing of intervention rather than cyst size or duration alone. A multidisciplinary, patient-centered strategy remains essential for achieving optimal outcomes while minimizing unnecessary intervention.

## Introduction and background

Introduction

Pancreatic pseudocysts represent one of the most common local complications of pancreatitis and continue to pose diagnostic and therapeutic challenges in clinical practice. A pancreatic pseudocyst is defined as a localized collection of pancreatic fluid enclosed by a wall of fibrous or granulation tissue, lacking an epithelial lining, and typically developing as a sequela of acute pancreatitis, chronic pancreatitis, or pancreatic trauma. Although many pseudocysts follow a benign course, others may progress to cause significant morbidity through infection, hemorrhage, rupture, or the compression of adjacent organs [1].

Historically, pancreatic pseudocysts were managed aggressively with surgical drainage based on early observations that cysts persisting beyond six weeks or exceeding 6 cm in diameter were unlikely to resolve spontaneously [2]. This size- and time-based paradigm has since been challenged. Subsequent prospective and observational studies demonstrated that size alone is an unreliable predictor of outcome and that many pseudocysts, particularly those arising after acute pancreatitis, may resolve with conservative management [3]. These insights, combined with advances in diagnostic imaging and minimally invasive interventions, have resulted in a paradigm shift toward individualized, evidence-based management.

The modern management of pancreatic pseudocysts relies on accurate classification, the careful assessment of symptoms and complications, and the selection of an appropriate treatment modality based on anatomy and disease biology. Endoscopic and image-guided approaches now offer outcomes comparable to surgery in selected patients, with lower morbidity and shorter hospital stay [4]. Despite these advances, variability persists in clinical decision-making, underscoring the need for a structured and comprehensive review of current evidence.

Definitions and classification

The accurate classification of pancreatic fluid collections is fundamental to appropriate management. The Revised Atlanta Classification provides a standardized framework for categorizing pancreatic and peripancreatic collections based on timing, contents, and wall maturity [5]. Cross-sectional imaging plays a critical role in applying this classification in clinical practice, particularly in distinguishing simple fluid collections from those containing necrotic debris, which has direct implications for treatment selection [6].

According to this classification, a pancreatic pseudocyst is a mature, encapsulated collection containing predominantly fluid, usually developing more than four weeks after the onset of pancreatitis. This entity must be clearly distinguished from acute peripancreatic fluid collections, which lack a defined wall and occur early in the disease course, as well as from collections containing necrotic material, such as acute necrotic collections and walled-off necrosis.

This distinction is clinically critical. Pancreatic pseudocysts are generally amenable to simple drainage procedures, whereas necrotic collections often require more complex interventions, including endoscopic or surgical necrosectomy, and are associated with higher complication rates [6]. Failure to correctly classify a pancreatic fluid collection may lead to inappropriate treatment and adverse outcomes.

According to the Revised Atlanta Classification, pancreatic and peripancreatic fluid collections are categorized based on timing, contents, and wall maturity, which has direct implications for management strategies [5,6]. The key distinguishing features of these entities are summarized in Table 1.

**Table 1:** Revised Atlanta Classification of Pancreatic and Peripancreatic Fluid Collections Adapted from Banks et al. (2013) [5]

Collection Type	Time From Onset	Contents	Wall	Clinical Implication
Acute peripancreatic fluid collection	<4 weeks	Fluid	Absent	Usually resolves
Pancreatic pseudocyst	≥4 weeks	Fluid	Present	Suitable for drainage if symptomatic
Acute necrotic collection	<4 weeks	Necrotic tissue ± fluid	Absent	Intervention usually delayed
Walled-off necrosis	≥4 weeks	Necrotic debris ± fluid	Present	Often requires necrosectomy

Epidemiology and natural history

The incidence of pancreatic pseudocysts varies according to the underlying pancreatic pathology. Following acute pancreatitis, pseudocysts develop in approximately 5%-16% of patients, whereas in chronic pancreatitis, the incidence may reach 20%-40% [7]. Alcohol-related pancreatitis and long-standing chronic pancreatitis are particularly associated with pseudocyst formation due to persistent ductal injury and increased intrapancreatic pressure.

Natural history studies have shown that a substantial proportion of pseudocysts resolve spontaneously, particularly those arising after acute pancreatitis. In a landmark prospective study, Yeo et al. demonstrated that asymptomatic pseudocysts frequently regress with conservative management [3]. Subsequent studies confirmed that symptomatology and complications, rather than cyst size alone, are the most reliable predictors of outcome [8].

In contrast, pseudocysts associated with chronic pancreatitis are less likely to resolve spontaneously. Ongoing ductal obstruction and fibrosis promote persistence and recurrence, often necessitating intervention. Prolonged observation in symptomatic patients increases the risk of complications such as infection, hemorrhage, and nutritional compromise [7].

Pathophysiology

Pancreatic pseudocysts develop as a consequence of pancreatic ductal disruption with the leakage of enzyme-rich pancreatic secretions into surrounding tissues. In acute pancreatitis, ductal injury is often transient and related to inflammatory necrosis. Over time, the extravasated fluid becomes encapsulated by fibrous and granulation tissue, forming a pseudocyst [9].

In chronic pancreatitis, persistent ductal strictures, intraductal calculi, and parenchymal fibrosis lead to sustained intraductal hypertension and recurrent leakage. This explains the higher incidence, persistence, and recurrence of pseudocysts in chronic disease. Importantly, communication between the pseudocyst and the pancreatic duct significantly influences both natural history and treatment outcomes [9].

Pseudocysts associated with complete ductal disruption, as seen in disconnected pancreatic duct syndrome (DPDS), rarely resolve with simple drainage procedures and often require long-term internal drainage or definitive surgical management [10].

Clinical presentation

The clinical manifestations of pancreatic pseudocysts depend on size, location, the rate of expansion, and the presence of complications. Abdominal pain is the most common presenting symptom and may result from pancreatic inflammation, capsular tension, or the compression of adjacent structures [7].

Large pseudocysts may cause early satiety, nausea, vomiting, or weight loss due to gastric or duodenal compression. The compression of the distal common bile duct may result in obstructive jaundice. Infected pseudocysts typically present with fever, leukocytosis, and systemic inflammatory response [8].

Life-threatening complications include hemorrhage, often due to erosion into adjacent vessels with pseudoaneurysm formation, and rupture into the peritoneal cavity, leading to peritonitis and shock. The recognition of these presentations is critical, as they mandate urgent intervention [8].

The clinical manifestations of pancreatic pseudocysts vary depending on size, location, and the presence of complications. Common symptoms and their underlying mechanisms, along with their clinical significance, are summarized in Table 2 [7,8].

**Table 2:** Clinical Presentations and Complications of Pancreatic Pseudocysts Author-generated table based on synthesized literature

Feature	Mechanism	Clinical Significance
Abdominal pain	Capsular tension and inflammation	Most common symptom
Early satiety/vomiting	Gastric or duodenal compression	Indication for intervention
Jaundice	Biliary compression	Suggests a large head-region cyst
Infection	Bacterial contamination	Requires drainage
Hemorrhage	Pseudoaneurysm erosion	Life-threatening emergency
Rupture	Wall failure	Surgical emergency

Diagnostic evaluation

The accurate diagnosis of pancreatic pseudocysts requires the integration of clinical history, biochemical parameters, and imaging findings. A prior episode of acute pancreatitis, chronic pancreatitis, or pancreatic trauma strongly supports the diagnosis. However, the absence of a documented pancreatitis episode does not exclude a pseudocyst, particularly in patients with alcohol-related or subclinical chronic pancreatitis.

Laboratory investigations are nonspecific but may provide supportive information. Serum amylase and lipase levels are often normal by the time a pseudocyst has matured. Elevated inflammatory markers suggest infection, while deranged liver function tests may indicate biliary obstruction secondary to extrinsic compression. Importantly, biochemical markers alone are insufficient to distinguish pseudocysts from cystic pancreatic neoplasms, necessitating high-quality imaging and, in selected cases, endoscopic evaluation [11].

A critical diagnostic objective is to differentiate pancreatic pseudocysts from other pancreatic fluid collections and cystic lesions, as management strategies differ substantially. Misclassification may result in the inappropriate drainage of necrotic collections or the delayed diagnosis of cystic neoplasms.

Imaging modalities

Contrast-Enhanced Computed Tomography (CECT)

Contrast-enhanced computed tomography (CT) remains the cornerstone of initial evaluation for pancreatic pseudocysts. It provides essential information regarding cyst size, location, wall thickness, internal contents, and relationship to adjacent organs and vascular structures [12]. CT is particularly useful for detecting complications such as hemorrhage, rupture, infection, and biliary or gastrointestinal obstruction.

Mature pseudocysts typically appear as well-defined, homogeneous, low-attenuation lesions with a smooth, enhancing wall. The presence of internal debris, irregular margins, or gas suggests infection or necrosis and warrants further evaluation. CT angiography is invaluable when hemorrhage is suspected, as it may identify pseudoaneurysms requiring urgent intervention [13].

*Magnetic Resonance Imaging and Magnetic Resonance* *Cholangiopancreatography (MRI/MRCP)*

MRI provides superior soft-tissue contrast and is particularly useful in characterizing cyst contents and assessing pancreatic duct anatomy. MRCP allows the noninvasive visualization of the pancreatic duct and can identify ductal disruptions or communication with the pseudocyst [14].

MRI is especially valuable when the diagnosis is uncertain or when differentiation from cystic pancreatic neoplasms is required. High signal intensity on T2-weighted images and the absence of enhancing mural nodules favor a pseudocyst, whereas complex septations or nodularity raise suspicion for neoplastic pathology.

Endoscopic Ultrasound (EUS)

Endoscopic ultrasound plays a pivotal role in both diagnosis and management. EUS offers high-resolution imaging of cyst wall characteristics, internal contents, and surrounding vasculature. The use of Doppler imaging allows the identification of intervening vessels, significantly reducing the risk of bleeding during transmural drainage [15].

EUS-guided fine-needle aspiration may be considered in selected cases where differentiation from cystic neoplasms is required. The analysis of cyst fluid amylase, carcinoembryonic antigen (CEA), and cytology can aid diagnosis, although routine aspiration is not recommended for typical pseudocysts due to infection risk [11].

Multiple imaging modalities are used in the evaluation of pancreatic pseudocysts, each with distinct advantages and limitations. Their comparative roles in diagnosis and management are outlined in Table 3 [12-15].

**Table 3:** Imaging Modalities in the Evaluation of Pancreatic Pseudocysts Author-generated table based on multiple sources CECT, contrast-enhanced computed tomography; MRI, magnetic resonance imaging; MRCP, magnetic resonance cholangiopancreatography; EUS, endoscopic ultrasound; PCD, percutaneous catheter drainage

Modality	Key Advantages	Limitations	Clinical Role
CECT	Widely available and detects complications	Limited ductal detail	First-line imaging
MRI/MRCP	Superior soft-tissue contrast and ductal anatomy	Cost and availability	Problem-solving and ductal assessment
EUS	High resolution and therapeutic capability	Invasive and operator-dependent	Diagnostic confirmation and drainage planning
Ultrasound	Bedside availability	Limited by bowel gas	Follow-up and guidance for PCD

## Review

Methodology

This review was designed as a comprehensive narrative synthesis of the published literature on pancreatic pseudocysts, with the objective of integrating evidence on disease mechanisms, diagnostic strategies, and contemporary management options. A structured literature search was conducted using PubMed and Scopus. Accepted indications for intervention include persistent or worsening abdominal pain, evidence of gastric outlet or biliary obstruction, infection, hemorrhage or pseudoaneurysm formation, and rupture. The progressive enlargement of the pseudocyst or failure to resolve after an adequate period of observation also warrants active treatment. Importantly, decision-making should be guided by clinical presentation and complications rather than cyst size alone [16,17].

Importantly, cyst size alone is no longer considered an absolute indication for intervention. Rather, symptomatology and complications guide decision-making [18].

The accepted indications for intervention are based on symptomatology, complications, and disease progression rather than cyst size alone. These indications and their clinical rationale are summarized in Table 4 [18].

**Table 4:** Indications for Intervention in Pancreatic Pseudocysts Author-generated summary of commonly accepted clinical indications

Indication	Rationale
Persistent pain	Reduced quality of life
Obstruction	Risk of malnutrition and jaundice
Infection	Risk of sepsis
Hemorrhage	Life-threatening complication
Rupture	Surgical emergency
Progressive enlargement	Risk of complications

Timing of intervention

The timing of intervention is critical to optimize outcomes. Drainage is generally deferred until at least four weeks after pancreatitis onset to allow the maturation of the cyst wall. Early intervention is reserved for urgent complications such as infection or hemorrhage.

Delayed intervention allows safer drainage, reduces recurrence, and minimizes procedural complications. The premature drainage of immature collections is associated with higher failure rates and morbidity.

Management of pancreatic pseudocyst: A stepwise, evolving therapeutic narrative

The management of pancreatic pseudocysts has undergone a remarkable evolution over the past four decades. What was once considered a surgical disease requiring mandatory operative drainage has gradually transformed into a condition managed through carefully staged, minimally invasive strategies. This shift reflects a deeper understanding of disease biology, improved imaging, and the emergence of advanced endoscopic and interventional radiological techniques. Yet, despite these advances, no single treatment modality suits all patients. Successful management depends on choosing the right intervention for the right pseudocyst at the right time.

Conservative management: When doing less is doing right

The story of pancreatic pseudocyst management often begins with restraint. Not every pseudocyst demands intervention, and premature treatment may expose patients to unnecessary risk. Observational studies have consistently shown that asymptomatic pseudocysts, particularly those arising after acute pancreatitis, may resolve spontaneously as inflammation subsides and ductal integrity is restored.

Conservative management is appropriate in patients who are asymptomatic, hemodynamically stable, and free of complications such as infection, bleeding, or obstruction. These patients are managed with analgesia, nutritional optimization, abstinence from alcohol, and close clinical and radiological surveillance. Serial imaging at 4-6-week intervals allows the assessment of cyst evolution and wall maturation [19].

However, observation has limits. Persistence beyond six weeks, progressive enlargement, or the development of symptoms signals the need for active intervention. Conservative management should therefore be viewed as a deliberate therapeutic phase, not indecision.

Endoscopic management: The modern first line

Endoscopic drainage has emerged as the preferred initial intervention for most symptomatic pancreatic pseudocysts, provided anatomical criteria are met. The introduction of endoscopic ultrasound (EUS) transformed this field, shifting drainage from a blind, risk-laden procedure to a precise, image-guided intervention.

EUS-Guided Transmural Drainage

EUS-guided cystogastrostomy or cysto-duodenostomy is now considered the standard of care for mature pseudocysts that lie in close apposition to the stomach or duodenum [20]. Under EUS guidance, the pseudocyst wall is punctured after the Doppler confirmation of an avascular tract, followed by the dilation and placement of internal stents.

Two stent strategies dominate contemporary practice. Plastic double-pigtail stents have a long safety record and are effective for simple, fluid-filled pseudocysts. Lumen-apposing metal stents (LAMS), by contrast, provide a large-caliber conduit that allows rapid drainage and endoscopic access if solid debris is present [21].

Comparative studies and meta-analyses have shown that EUS-guided drainage achieves technical success rates exceeding 85%-90%, with clinical success comparable to surgical drainage but with shorter hospital stay, lower cost, and reduced morbidity [22].

Yet, endoscopic success depends on discipline. Inappropriate stent choice, failure to remove LAMS in a timely fashion, or the drainage of collections with significant necrosis may lead to bleeding, stent burial, or infection [23].

Trans-papillary (Endoscopic Retrograde Cholangiopancreatography {ERCP}-Guided) Drainage

Trans-papillary drainage occupies a more selective role. This approach targets the underlying ductal pathology rather than the cyst itself. When a pseudocyst communicates directly with the pancreatic duct, particularly in chronic pancreatitis, the placement of a pancreatic duct stent can decompress the system and promote cyst resolution [24].

Trans-papillary drainage is rarely sufficient as monotherapy for large pseudocysts but serves as a valuable adjunct to transmural drainage. Its limitations become evident in the presence of complete ductal disruption, where pancreatic secretions continue to leak unabated.

Percutaneous Drainage: The Bridge, Not the Destination

Percutaneous catheter drainage (PCD) remains an important tool, particularly in unstable patients or those with infected pseudocysts. Performed under ultrasound or CT guidance, PCD provides rapid source control and can be lifesaving in patients with sepsis [25].

However, percutaneous drainage comes at a cost. External pancreatic fistula, prolonged catheter dependence, and higher recurrence rates are well-documented limitations. Comparative analyses consistently show higher re-intervention rates compared with endoscopic or surgical drainage [26].

For this reason, PCD is best viewed as a bridging strategy, a means to stabilize the patient before definitive internal drainage or surgery, rather than a definitive solution in most cases.

Surgical management: The enduring backbone

Despite the rise of minimally invasive techniques, surgery retains a central role in the management of pancreatic pseudocysts. Surgical intervention offers the most durable solution and remains indispensable in complex or refractory disease.

Surgical intervention is indicated in cases of failed endoscopic or percutaneous drainage, multiloculated or large pseudocysts, and the presence of disconnected pancreatic duct syndrome. It is also required when pseudocysts are associated with underlying pancreatic pathology necessitating resection or when complications such as rupture or uncontrolled hemorrhage occur [27].

Surgical Techniques

Internal drainage procedures, cystogastrostomy, cysto-duodenostomy, or cystojejunostomy, establish a wide, permanent communication between the pseudocyst and the gastrointestinal tract. The choice of procedure depends on cyst location and anatomy.

Laparoscopic approaches have largely replaced open surgery in suitable candidates, offering comparable success with reduced postoperative pain, shorter hospital stay, and faster recovery [28]. In selected patients, pancreatic resection may be required, particularly when pseudocysts coexist with irreversible chronic pancreatitis or neoplasia.

The various management strategies for pancreatic pseudocysts differ in their indications, advantages, and limitations. A comparative overview of these approaches is provided in Table 5 [20-26].

**Table 5:** Comparative Overview of Treatment Modalities Author-generated table based on published comparative studies EUS: endoscopic ultrasound

Modality	Ideal Indication	Advantages	Limitations
Conservative	Asymptomatic, small cysts	Avoids intervention	Risk of persistence
Endoscopic (EUS)	Symptomatic, mature cysts	Minimally invasive	Requires expertise
Percutaneous	Infected/unstable patients	Rapid source control	External fistula
Surgical	Complex or refractory disease	Durable cure	Higher morbidity

Disconnected pancreatic duct syndrome: The turning point

Disconnected pancreatic duct syndrome (DPDS) represents a critical inflection point in pseudocyst management. In this condition, a segment of the pancreatic duct is completely disconnected, usually following necrotizing pancreatitis. Simple drainage, endoscopic or percutaneous, fails because pancreatic secretions continue to flow from the isolated gland [29].

Definitive management requires long-term internal drainage or surgical resection. Failure to recognize DPDS early often results in repeated unsuccessful interventions, prolonged morbidity, and patient frustration.

## Conclusions

Pancreatic pseudocysts are increasingly encountered in clinical practice and encompass a wide spectrum of disease behavior. Management has evolved from routine surgical drainage to a more selective, individualized approach guided by symptoms, complications, and underlying pancreatic duct anatomy. Advances in imaging and minimally invasive techniques have improved diagnostic accuracy and expanded therapeutic options, allowing many patients to be treated effectively without surgery.

A structured evaluation incorporating clinical assessment and appropriate imaging is essential to distinguish pseudocysts from other pancreatic cystic lesions and to guide treatment planning. While many asymptomatic pseudocysts can be managed conservatively with surveillance, intervention is required in the presence of symptoms or complications. Endoscopic approaches are now the preferred first-line modality in suitable cases, with percutaneous and surgical options reserved for specific clinical scenarios. Optimal outcomes depend on careful patient selection, the appropriate timing of intervention, and a multidisciplinary approach. Tailoring management to individual disease characteristics remains the cornerstone of safe and effective treatment.
